# Coronary Artery Ectasia: Case Reports and Literature Review

**DOI:** 10.7759/cureus.62011

**Published:** 2024-06-09

**Authors:** Mouad Lamtai, Youssef Fihri, Soumia Faid, Laktib Nabil, Driss Britel, Hicham Faliouni, Najat Mouine, Zouhair Lakhal, Aatif Benyass

**Affiliations:** 1 Cardiology Center, Military Hospital Mohamed V, Rabat, MAR

**Keywords:** myocardial infarction, novel oral anticoagulation, coronary angiography, abnormal dilatation, coronary artery

## Abstract

Coronary artery ectasia (CAE) is a rare condition, affecting 3%-8% of patients with atherosclerotic coronary artery disease, and is characterized by the abnormal dilatation of the coronary arteries. While the etiology of coronary artery ectasia encompasses a myriad of acquired and genetic factors, its pathogenesis still remains a subject of investigation.

The clinical manifestations are varied, ranging from asymptomatic cases to chest angina and myocardial infarction. Coronary angiography remains the gold standard for diagnosing CAE.

We herein report four cases of coronary ectasia: the first involving myocardial infarction, the second associated with bicuspid aortic valve with severe aortic regurgitation, the third detected during coronary angiography for moderate left ventricular dysfunction, and the last one detected during coronary angiography for stable angina.

The aims of our study are to highlight the diversity of clinical presentations as well as the challenge of management, given that there are no universal treatments or guidelines.

## Introduction

Coronary artery ectasia (CAE) or aneurysmal coronary artery disease is characterized by the abnormal dilatation of a coronary artery, where the dilated segment has a diameter more than 1.5 times the diameter of a normal adjacent segment [1]. When the dilatation affects only a specific coronary segment, it's termed as coronary artery aneurysm, while it's referred to as coronary artery ectasia when it involves the entire vessel [2]. The mechanisms underlying its pathophysiology are still unclear, but they are mostly attributed to atherosclerosis in 50% of cases [3].

We will be presenting four cases of coronary artery ectasia and discussing their etiology, complications, diagnostic modalities, and available therapeutic interventions.

## Case presentation

Case 1

A 54-year-old man was hospitalized in the emergency department at the 4th hour of a severe, prolonged constrictive chest pain. He had a high cardiovascular risk: type 2 diabetes, hypertension and dyslipidemia. He had never smoked and had no personal or family history of cardiovascular disease. He also reported having two weeks of increasing exertional chest pain radiating to the left arm.

The physical exam showed a normal heart rate (79 beats per minute), blood pressure (141/84 mmHg) and normal cardiac sounds with no signs of heart failure.

An 18-lead electrocardiogram (EKG) derivation was performed and revealed sinus rhythm with ST elevation in the inferior and posterior leads (II, aVF, V7, V8, V9) with anterior ST depression, suggesting an inferior and posterior myocardial infarction. The initial serum troponin level was 9166 ng/mL (Normal value is 16 ng/ml).

**Figure 1:**
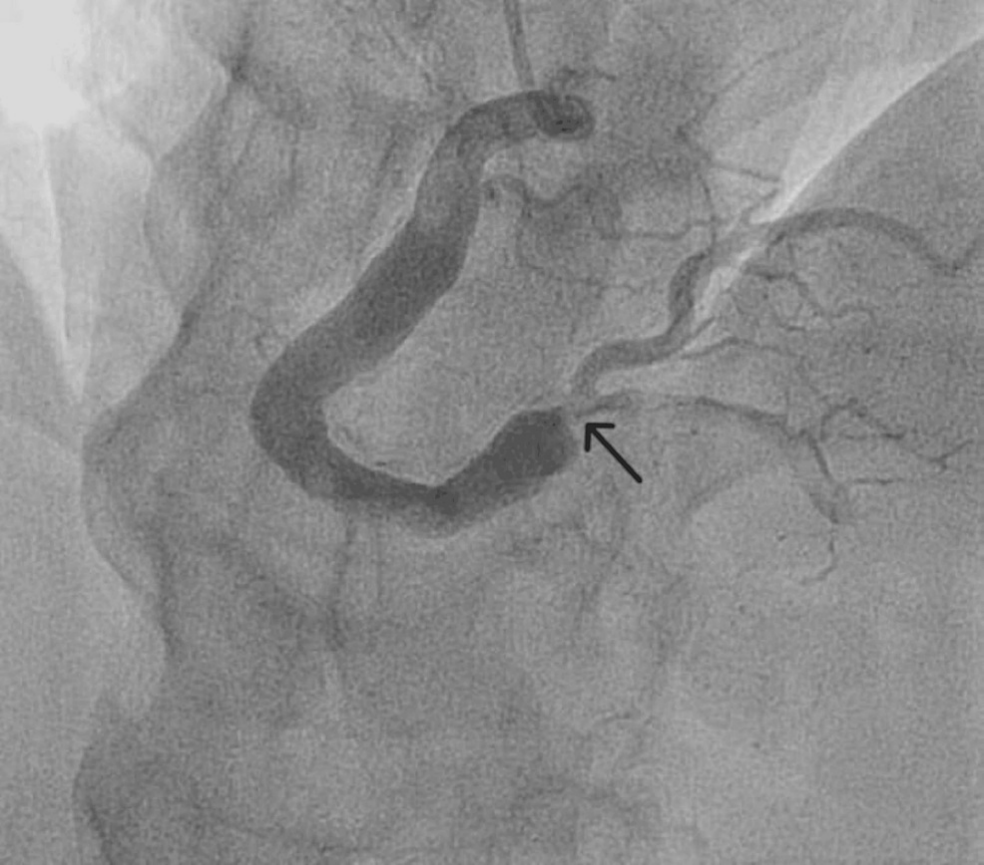
Ectatic right coronary artery with distal segment occlusion (black arrow).

**Figure 2:**
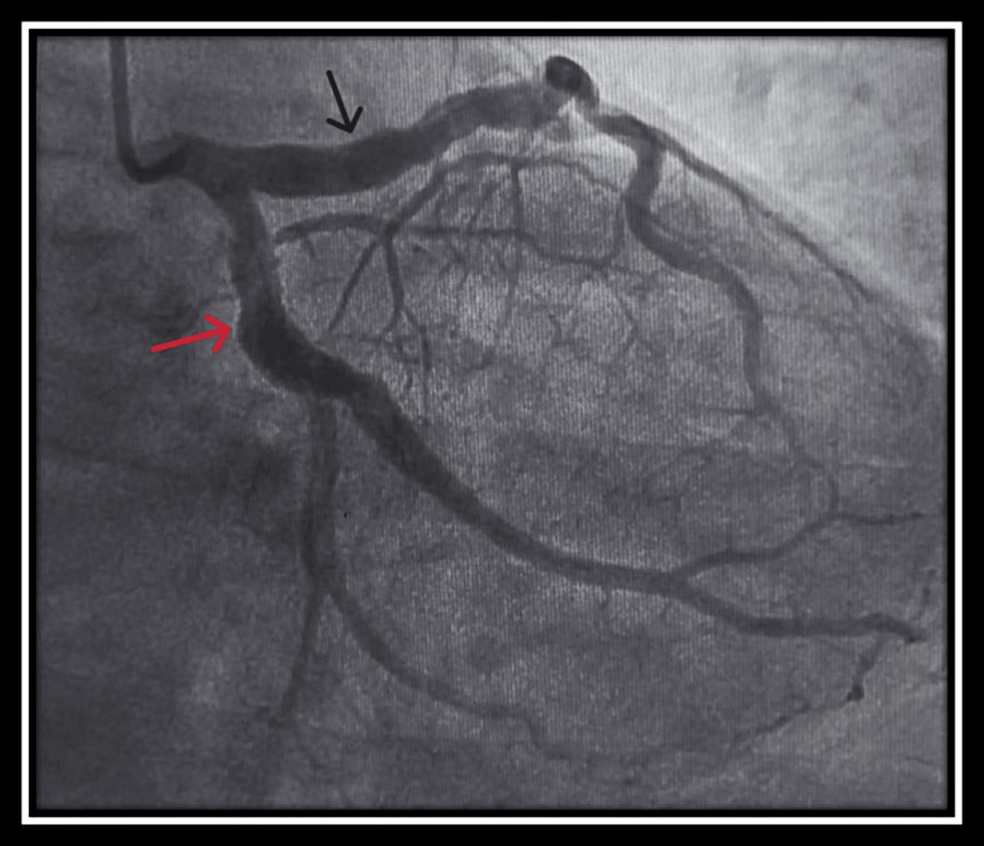
Ectasia of the proximal part of the left anterior descending artery (black arrow) and circumflex coronary artery (red arrow) without a significant stenosis.

Emergency coronary angiography showed that the right coronary artery was ectatic with total occlusion of the distal segment and suspicion of a thrombus (Figure 1). The left anterior descending artery and circumflex coronary artery were also ectatic, particularly in the proximal part, without significant stenosis (Figure 2).

Owing to concerns that the presence of thrombus would predispose the patient to a high risk of stent thrombosis and the risk of stent malposition, the patient received optimal medical therapy based on unfractionated heparin followed by dual antiplatelet therapy (aspirin and clopidogrel), an angiotensin-converting enzyme inhibitor, a beta blocker, a statin, and novel oral anticoagulation (Rivaroxaban).

Two years later, the patient remained asymptomatic, with no recurrence of angina pectoris. A follow-up echocardiogram revealed moderate left ventricular dysfunction accompanied by wall abnormalities.

Case 2

A 50-year-old man with a history of uncontrolled hypertension, presented at the emergency department with progressive shortness of breath and paroxysmal nocturnal dyspnea.

Physical examination revealed blood pressure at 146/45 mmHg, the point of maximal impulse displaced inferiorly and laterally, a decrescendo early-diastolic blowing murmur, and a positive Musset’s sign (a rhythmic nodding of the head in synchrony with the beating of the heart).

The EKG showed sinus rhythm with a complete left bundle branch block and multiple premature ventricular contractions. Cornell criteria for left ventricular hypertrophy were positive.

**Figure 3:**
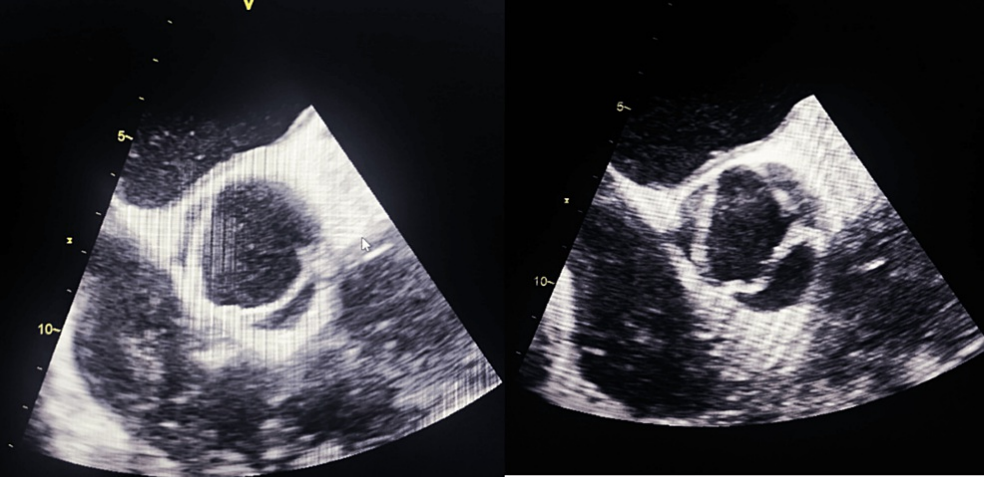
Transesophageal echocardiography showing left-right type 1 bicuspid aortic valve.

**Figure 4:**
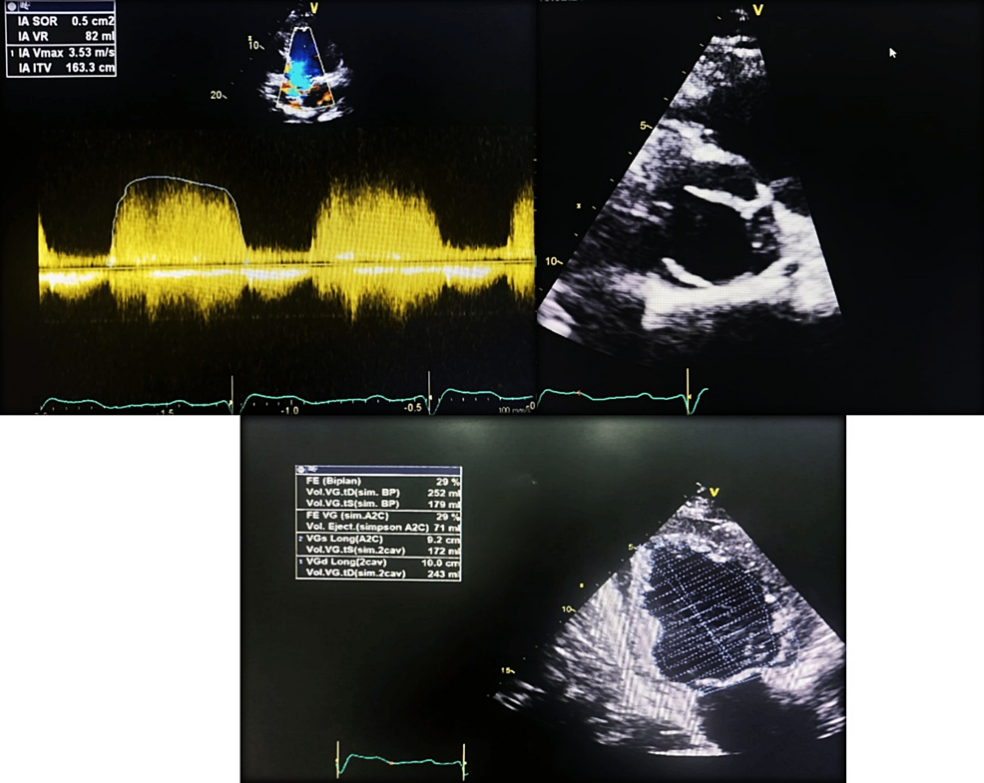
Transthoracic echocardiography showing bicuspid aortic valve with severe aortic regurgitation and left ventricular dysfunction.

Transthoracic and transesophageal echocardiography revealed severe aortic regurgitation with a left-right type 1 bicuspid aortic valve without dilatation of the ascending aorta and severe left ventricular dysfunction (LVEF 29%) (Figure 3, Figure 4).

**Figure 5:**
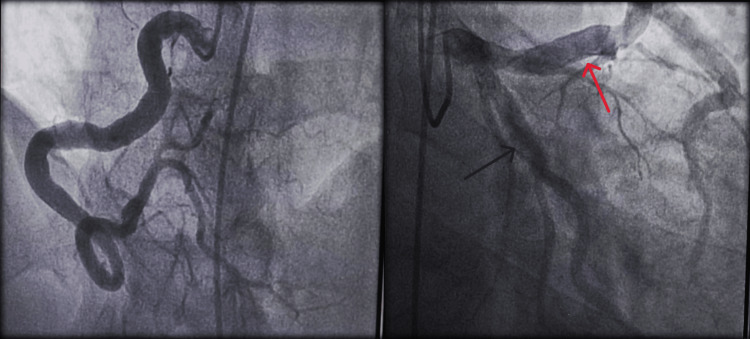
Left picture: right coronary artery; Right picture: left anterior descending artery (red arrow) and circumflex coronary artery (black arrow).

Pre-operative coronary angiography showed multiple coronary segments with coronary slow-flow phenomenon (CSFP) (Figure 5).

He was transferred to the cardiac surgery department, where he underwent aortic valve replacement surgery.

Case 3

A 67-year-old man was referred to our hospital for further evaluation. He was asymptomatic until one month before admission when he began experiencing dyspnea on exertion. He was recently diagnosed with diabetes. He also denied any chest pain or angina equivalent.

His physical exam was normal. His EKG showed a normal sinus rhythm with left axial deviation, left anterior fascicular block and 1st-degree block.

He underwent a transthoracic echocardiogram, which showed dilated left ventricular size with wall motion abnormalities with LVEF of 40%. There was no significant valvular heart disease.

**Figure 6:**
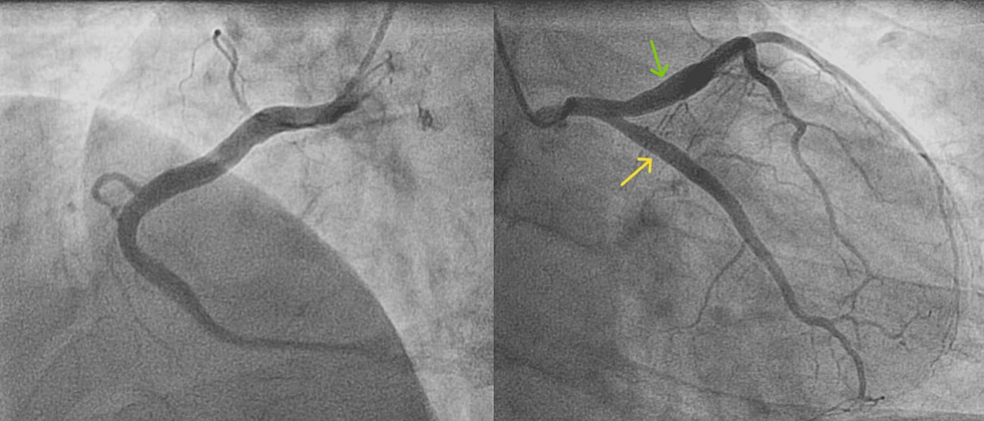
Multiple ectatic coronary segments: Left picture: right coronary artery; Right picture: left anterior descending artery (green arrow) and circumflex coronary artery (yellow arrow).

To rule out coronary artery disease, the patient underwent coronary angiography. It revealed no significant stenosis in the coronary arteries, but the right coronary artery (RCA), circumflex coronary artery (LCX), and left anterior descending artery (LAD) were all large luminal vessels with a slow flow of contrast (Figure 6).

The patient was treated with aspirin, a beta blocker, a statin, an SGLT2 inhibitor (dapagliflozin) and a novel oral anticoagulation (rivaroxaban). A cardiac magnetic resonance (CMR) was planned for further investigation of his ventricular dysfunction.

Case 4

A 51-year-old man presented with a history of typical chest angina. He also has a history of severe hypertension secondary to adrenal adenoma for which he underwent adrenalectomy. His EKG indicated left ventricular hypertrophy. He underwent a dobutamine stress echocardiogram, which was positive (ST elevation of 2 mm in aVR derivation, with ST depression in leads V1 to V6, along with akinesia in the inferoseptal and inferior regions, and hypokinesia in the basal and mid segments of the anteroseptal wall).

**Figure 7:**
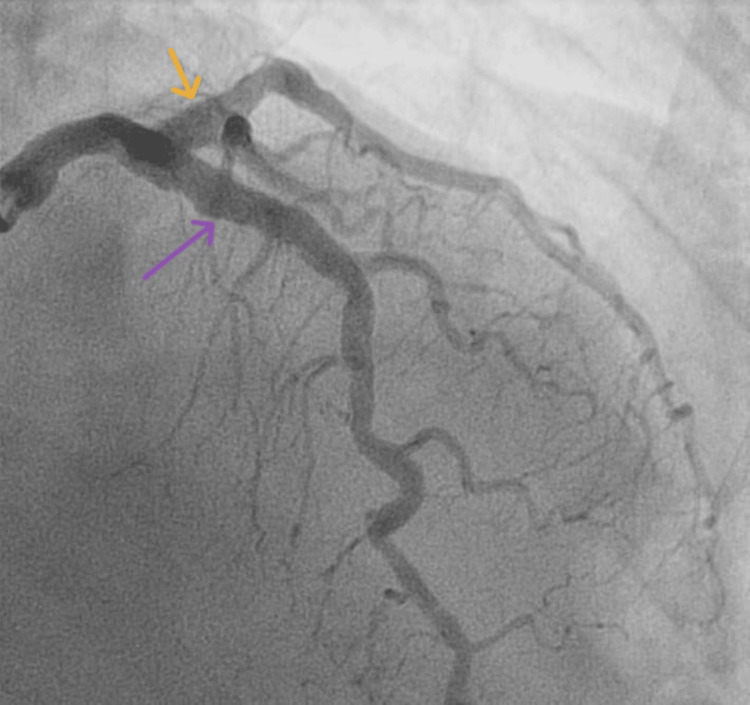
Ectasia of the proximal part of the left anterior descending artery (orange arrow) and the proximal left circumflex artery (violet arrow).

Coronary angiography showed an ectasia of the proximal part of the left anterior descending artery, the proximal left circumflex artery (Figure 7) and normal right coronary artery without any stenosis.

The patient was treated with aspirin, a beta blocker, a statin and a novel oral anticoagulation (rivaroxaban).

He was instructed to return for a follow-up, showing improved clinical symptoms with no recurrent angina.

## Discussion

The definition of coronary ectasia implies the existence of dilatation with a diameter of 1.5 times the adjacent normal coronary artery [4].

There are four types of coronary artery ectasia [4]: Type 1 (diffuse ectasia of two or three coronary artery), type 2 (diffuse ectasia in one coronary artery and localized disease in another coronary artery), type 3 (diffuse ectasia in one coronary artery only), type 4 (localized or segmental involvement). Based on this classification our case reports are Type 1.

Coronary ectasia can develop as a result of acquired conditions like atherosclerosis (50%) or systemic diseases like Kawasaki, but it can also be congenital associated with other cardiac abnormalities such as bicuspid aortic valve [4]. According to a study in Germany, coronary ectasia occurred more than twice in people with bicuspid aortic valve compared with people with tricuspid aortic valve, suggesting that coronary ectasia could be related to genetic factors [5].

The clinical presentation can vary from one patient to another. Angina is the most common symptom caused by the slow coronary flow - even when there is no associated coronary artery stenosis -, an acute coronary syndrome occurring due to the development of intracoronary thrombus or dissipation of microemboli to distal coronary tree and it can also be asymptomatic detected incidentally during coronary angiography [6-7].

Coronary angiography stands as the foremost method for diagnosing coronary aneurysms, offering insights not just into their shape, size, location, and severity, but also into the potential existence of coronary stenosis [8]. CAE can also be diagnosed with noninvasive modalities such as coronary magnetic resonance angiography (MRA) and coronary artery computed tomography (CACT) [9].

There is currently no universal approach for managing coronary ectasia. Due to the involvement of atherosclerosis in the development of most coronary artery ectasia cases, it has been suggested to consider the administration of aspirin in all patients with CAE [7]. Decreased coronary flow velocity or stagnancy of coronary blood flow causes the formation of intracoronary thrombus, therefore a lot of studies have recommended the use of long-term oral anticoagulation based on Warfarin or novel oral anticoagulants [10-11]. Lipid-lowering medications such as statins can also be used due to their anti-inflammatory properties [6]. Hypertension is one of the main risk factors of coronary ectasia, therefore the use of anti-hypertensive treatment such as ACE inhibitors is justified since they may prevent the progression of coronary dilation by reducing intramural pressure [6]. The use of beta-blockers is a matter of debate because they can lower myocardial oxygen demand but also increase the risk of vasospasm. Calcium channel blockers can be used for their anti-hypertensive and anti-spasm effects [6].

When dealing with acute myocardial infarction, performing percutaneous coronary intervention on an ectatic culprit vessel has consistently shown lower procedural success rates. Additionally, there's a higher incidence of no-reflow and distal embolization, along with increased rates of subsequent stent thrombosis, repeat revascularization, and long-term mortality. This can be explained by residual thrombus, turbulent flow, and the high risk of stent malposition [12-13]. In acute myocardial infarction, coronary blood flow can also be restored by medical therapy with heparin infusion as well as thrombolysis [2-15].

## Conclusions

Coronary artery ectasia is an uncommon finding during coronary angiography, characterized by abnormal dilatation of coronary arteries. Atherosclerosis remains the principal etiologic cause. The severity and progression of symptoms can vary among patients, ranging from asymptomatic cases to myocardial infarction. Managing coronary ectasia presents clinical challenges due to its variable clinical presentations and associated complications. Hence, further studies are required to enhance our comprehension of the pathophysiology, identify risk profiles, and establish universal management guidelines.
